# Online Closed-Loop Control Using Tactile Feedback Delivered Through Surface and Subdermal Electrotactile Stimulation

**DOI:** 10.3389/fnins.2021.580385

**Published:** 2021-02-18

**Authors:** Jian Dong, Winnie Jensen, Bo Geng, Ernest Nlandu Kamavuako, Strahinja Dosen

**Affiliations:** ^1^Department of Orthopedics, The Second Hospital of Jilin University, Changchun, China; ^2^Department of Health Science and Technology, Aalborg University, Aalborg, Denmark; ^3^Centre for Robotics Research, Department of Informatics, King’s College London, London, United Kingdom

**Keywords:** closed-loop control, electrical stimulation, subdermal electrodes, prostheses, sensory feedback

## Abstract

**Aim:**

Limb loss is a dramatic event with a devastating impact on a person’s quality of life. Prostheses have been used to restore lost motor abilities and cosmetic appearance. Closing the loop between the prosthesis and the amputee by providing somatosensory feedback to the user might improve the performance, confidence of the amputee, and embodiment of the prosthesis. Recently, a minimally invasive method, in which the electrodes are placed subdermally, was presented and psychometrically evaluated. The present study aimed to assess the quality of online control with subdermal stimulation and compare it to that achieved using surface stimulation (common benchmark) as well as to investigate the impact of training on the two modalities.

**Methods:**

Ten able-bodied subjects performed a PC-based compensatory tracking task. The subjects employed a joystick to track a predefined pseudorandom trajectory using feedback on the momentary tracking error, which was conveyed via surface and subdermal electrotactile stimulation. The tracking performance was evaluated using the correlation coefficient (CORR), root mean square error (RMSE), and time delay between reference and generated trajectories.

**Results:**

Both stimulation modalities resulted in good closed-loop control, and surface stimulation outperformed the subdermal approach. There was significant difference in CORR (86 vs 77%) and RMSE (0.23 vs 0.31) between surface and subdermal stimulation (all *p* < 0.05). The RMSE of the subdermal stimulation decreased significantly in the first few trials.

**Conclusion:**

Subdermal stimulation is a viable method to provide tactile feedback. The quality of online control is, however, somewhat worse compared to that achieved using surface stimulation. Nevertheless, due to minimal invasiveness, compactness, and power efficiency, the subdermal interface could be an attractive solution for the functional application in sensate prostheses.

## Introduction

The loss of a hand through amputation has a devastating impact on the affected persons. Since the hand is an essential instrument in daily life activities, both the interaction with the environment and social communication are impaired. Prosthetic systems have been developed and used to replace the missing limb so that the motor functions are restored and thereby the quality of life of amputees is improved (Vujaklija et al., 2016). Currently, the most advanced devices are myoelectric prostheses, in which muscle electrical activity is recorded using electromyography (EMG) and translated into prosthesis commands (Parker et al., 2006; Fougner et al., 2012). There are many commercial prostheses with different numbers of active degrees of freedom, from simple grippers [e.g., Sensor Hand (Ottobock, 2020b)] to dexterous hands with individually controllable fingers [e.g., Bebionic hand (Ottobock, 2020a) and i-Limb (Ossur, 2020)]. However, none of them implement somatosensory feedback (feeling of touch) to the user, and therefore, the replacement of the hand functions is only partial (Biddiss et al., 2007; Kyberd et al., 2007). Only a few recently developed commercial prosthetic hands are advertized as providing sensory feedback (LUKE arm, 2020; Psyonic Ability Hand, 2020; VINCENTevolution 2, 2020), though, its clinical utility is still to be proven. Recent studies have demonstrated that closing the loop can improve performance (Clemente et al., 2016; Schiefer et al., 2016; Markovic et al., 2018; Shehata et al., 2018; Clemente et al., 2019; George et al., 2019; Petrini et al., 2019), compensate cognitive burden (Risso et al., 2019; Valle et al., 2020), and facilitate embodiment (Page et al., 2018; Valle et al., 2018a; Rognini et al., 2019). Therefore, the development of sensory feedback for myoelectric prostheses is an important task that is in the focus of the present research efforts (Sensinger and Dosen, 2020).

To close the loop, a prosthetic device needs to be sensorized. Supplemental feedback in a prosthesis can be provided using different methods to elicit tactile sensations. The sensor data are read, translated into stimulation profiles, which are then delivered to the natural sensory systems that are still available in the amputee user. For example, mechanical stimulation can be delivered to the skin of the residual limb using linear (Schoepp et al., 2018), rotational (Wheeler et al., 2010), or vibration motors (Witteveen et al., 2015; Engels et al., 2019; Pena et al., 2019), while electrical stimulation can be applied non-invasively to activate skin afferents and peripheral nerves (Szeto and Saunders, 1982; D’Anna et al., 2017; Osborn et al., 2018; Shin et al., 2018), and invasively to stimulate peripheral nerves (Raspopovic et al., 2014), or even brain (Tabot et al., 2013). The feedback information is transmitted by modulating stimulation parameters, e.g., the measured grasping force can be communicated through the intensity or frequency of stimulation (De Nunzio et al., 2017; Markovic et al., 2018).

Electrical stimulation is an appealing method for providing sensory feedback since the electronic stimulator is compact, power consumption is low, the response is fast since there are no mechanical components that need to be accelerated (as in, e.g., vibration motors) and the stimulation parameters (intensity and frequency) can be independently modulated. In vibration motors, on the contrary, the parameters are coupled through mechanical construction (e.g., mass-spring resonance). In invasive systems (Zollo et al., 2019), electrical stimulation is typically delivered by placing the electrodes around (cuff) (Tan et al., 2015; Graczyk et al., 2018) or inside (intraneural) peripheral nerves (Oddo et al., 2016; Valle et al., 2018a; George et al., 2019). In both cases, a surgical procedure is required, and some amputees, especially those that suffered traumatic injuries, might be reluctant to undergo additional surgical interventions (Geng et al., 2018). In non-invasive systems, the stimulation electrodes are placed on the surface of the skin. This is convenient, but the electrodes must be reapplied, and the electrode–skin interface has a high-impedance which is variable and unstable [e.g., sweating (Riso et al., 1989)].

Another possibility to apply electrical stimulation, which might combine good sides of both techniques (implanted and surface), is to use subdermal electrodes. They are inserted in the subdermal region of the skin using a hypodermic needle; hence, the procedure is minimally invasive, and yet, the electrodes avoid the high impedance of the skin layer. This approach has been proposed before (Riso et al., 1989), but it has not been thoroughly investigated. In recent studies, we have evaluated the sensation quality and psychometric properties of subdermal stimulation (Geng et al., 2018) as well as the stability of these parameters across hours (Dong et al., 2020b) and days (Dong et al., 2020a) and compared them to those of surface stimulation. The results demonstrated that subdermal electrical stimulation was able to produce similar sensation quality as surface stimulation while outperforming the latter in terms of energy efficiency (Geng et al., 2018). Nevertheless, in psychometric tests, the subject is a passive recipient of the stimulation, and he/she is only asked to verbally report on the sensation quality and quantity. Therefore, this assessment does not provide information on how useful subdermal stimulation is for online control.

The present study aimed to assess the quality of closed-loop control when subdermal and surface stimulation was used to provide tactile feedback on the ventral and dorsal side of the forearm. To this aim, the subjects were asked to perform a compensatory tracking task in which momentary tracking error was transmitted through the tactile feedback. Therefore, the subjects had not only to perceive the stimulation (as in psychometric testing) but also to interpret it and decide on the appropriate control action. The quality of online closed-loop control with subdermal tactile feedback was compared to that achieved using “conventional” surface stimulation (benchmark). The control has been tested across multiple trials to assess the impact of training on the two stimulation modalities.

## Materials and Methods

### Subjects

Ten able-bodied subjects (28.9 ± 2.7 years, six male, four female, nine right-handed) were recruited. The study was approved by the North Denmark Region Committee on Health Research Ethics (N-20160021) and conducted according to the Declaration of Helsinki. All subjects provided written informed consent.

### Experimental Procedure

#### Experimental Setup

The subject was seated in a comfortable chair facing a PC monitor placed on a table approximately 60 cm away. The middle areas of the volar and dorsal side of the forearm (dominant hand), one-third of the forearm length distally from the elbow, were gently shaved. The subdermal electrodes made of Teflon-coated stainless steel with the end 5 mm exposed (A-M Systems, Carlsborg, WA, United States, diameter 50 μm) were inserted just below the skin using a hypodermic needle, as explained in Geng et al. (2018). To prevent the electrode from moving, the wire was taped to the skin using medical tape. The surface self-adhesive electrodes (Ambu Neuroline 700, 20 mm × 15 mm) were then placed next to the insertion site of the subdermal electrode. The latter was inserted under an angle so that the tip of the wire was approximately below the surface electrode. One surface and one subdermal electrode were placed/inserted on each side of the forearm. The common ground electrode (PALS Platinum, 40 mm × 64 mm, oval) was positioned over the radial aspect of the wrist. The forearm of the subject was placed on the table so that he/she could hold the joystick in his/her hand comfortably. The joystick (APEM HF22X10U) allowed movement around two axes but only a single axis was used in the present experiment (one-dimensional tracking). The stimulation was delivered using a USB-powered stimulator (TremUNA, UNASystems, Serbia). TremUNA is a current-controlled stimulator that generates biphasic compensated electrical pulses. The stimulator integrates eight stimulation channels, but only two have been used in the present tracking study. The stimulation parameters can be adjusted independently for each channel. The ranges and increment/decrement steps for frequency are 0–50 Hz and 1 Hz, for pulse amplitude are 0–5 and 0.1 mA and for pulse width are 50–1,000 μs and 10 μs, respectively. The joystick and the stimulation unit were connected to the PC via a USB port. The stimulation was controlled online by sending text commands from the host PC to the stimulator via the USB port.

#### Experimental Task

**FIGURE 1:**
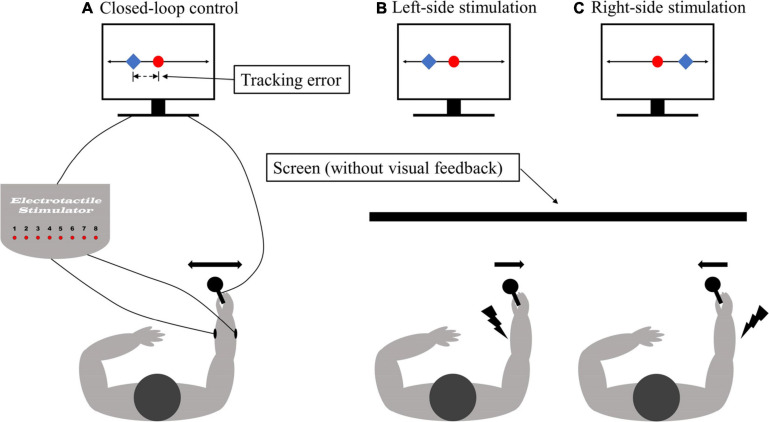
The illustration of the experimental setup. **(A)** The closed-loop control system for compensatory tracking using electrotactile feedback. The feedback was delivered via surface and subdermal electrodes placed on the dorsal and volar side of the forearm **(B,C)**. The task for the subject was to track a reference trajectory based on the feedback of the momentary tracking error. When using visual feedback, the tracking error was represented as the difference in position between the controlled cursor (blue square) and the stationary reference (red dot). For tactile feedback, the stimulation was presented to the ventral (dorsal) forearm when the controlled cursor moved to the left (right) of the reference position (red dot). In response, the subject moved the joystick to the right (left) to bring the cursor back to the reference. The subjects were blinded (without visual feedback) during the experiment trails.

The subject was asked to perform a one-dimensional compensatory tracking task using a joystick (see Figure 1), which is a commonly used experimental paradigm to evaluate human manual control (Mcruer and Weir, 1969; Dosen et al., 2014; Paredes et al., 2015). Therefore, the task for the subject was to track a reference trajectory relying on tactile feedback only (no visual feedback). Importantly, the target trajectory was not directly communicated to the subject but instead, the feedback provided a momentary deviation from the target trajectory (i.e., the tracking error). The subject moved the joystick to compensate for the tracking error (Figures 1B,C). With the visual feedback, which was used to introduce the task, the error was represented by a marker (blue, Figure 1), and the subject needed to move the joystick such that the blue marker remained over the static red target (zero error). With the electrotactile feedback, the error was conveyed through two electrodes. The dorsal electrode was active if the error was positive and oppositely, the volar electrode was active if the error was negative. The magnitude of the error was conveyed as the intensity of the stimulation adjusted by changing the pulse width. Therefore, the subject needed to move the joystick to minimize the intensity of the electrical stimulation (no stimulation = zero tracking error). The active electrode indicated the direction (left or right) in which the joystick should be moved to compensate for the error while the stimulation intensity was the cue for the size of this movement (e.g., larger error, stronger stimulation, and hence larger joystick movement). The online compensatory tracking task has been implemented using a test bench for human manual control (Dosen et al., 2015) based on Simulink Desktop Real-Time.

Each tracking trial lasted 90 s. The reference trajectory for the tracking was a pseudorandom multi-sine signal computed as a sum of nine sinusoids with randomized phases. The frequency of the sinusoids was selected by logarithmically dividing the range of frequencies between 0.01 and 0.5 Hz. The generated multi-sine was 30 s long, but this basic segment was then repeated three times resulting in a 90-s trial. The basic segment was long enough for the subject not to notice this repetition. A different signal was generated for each trial. For the subjects, therefore, the trajectory appeared to be random and impossible to predict. The amplitude of the trajectory was ±0.9 arbitrary units. The output of the joystick was also normalized (i.e., ±1 au). When using tactile feedback via electrical stimulation, the normalized tracking error was mapped linearly to the dynamic range of each subject: [0,1]⇒[*DT* + 0.1×*DR*,*PT*−0.1×*DR*], where *DT*, *PT*, and *DR* = *PT* − *DT* represent detection threshold, pain threshold, and dynamic range, respectively. Linear encoding is often used to implement tactile feedback in prostheses (Valle et al., 2018b; Mastinu et al., 2020). The mapping was selected so that the tactile feedback leads to a clear but not painful sensation.

#### Experimental Protocol

The *DT* and *PT* were determined for both surface and subdermal electrodes. The pulse amplitude was set to 3.5 and 3 mA in surface stimulation and 1.5 and 1 mA in subdermal stimulation for the dorsal and volar electrode, respectively. The frequency was fixed at 100 Hz throughout the experiment since this provides a clear and fused sensation that facilities closed-loop control (Paredes et al., 2015). The detection and pain thresholds were established using the method of limits (Kingdom and Prins, 2009). To this aim, the pulse width was increased from 50 μs in steps of 10 μs until the subject indicated that he/she felt the stimulation for the first time (*DT*). The increment step was then increased to 50 μs and the stimulation was delivered until the subject indicated that the sensation became uncomfortable. This procedure was repeated three times, and the average of the three values was adopted as the detection and pain threshold. If the muscle response was registered during the test, the location of the surface electrode was changed. If the muscle response could not be avoided, the stimulation amplitude leading to muscle activation was used as the upper threshold (instead of the pain threshold). Finally, in case *PT* could not be reached, the initial pulse amplitude was increased in steps of 0.5 mA and the aforementioned procedure was repeated.

Before starting the experimental trials, the subject first received training. They performed one trial of tracking using a simple sine wave with the visual feedback on the screen to understand the task. Then, they repeated the same task but this time with electrical stimulation delivered. Therefore, they could see the tracking error and feel the tactile sensations simultaneously. In this way, the subjects could associate the tracking error to the location (dorsal, volar) and intensity of the stimulation. After this, the subjects performed two trials where they tracked a pseudo-random multi-sine with visual feedback only and with simultaneous visual and electrotactile feedback. The aim was to train the tracking of a more complex trajectory. Finally, the subjects conducted eight experimental tracking trials only using tactile feedback. After each trial, they were informed about their performance to motivate them to improve their closed-loop control. The order of the two stimulation conditions was randomized across subjects.

### Outcome Measures

The outcome measures were the peak of the cross-correlation function (CORR), the root mean squared tracking error (RMSE), and the time delay (TD) between the generated and desired trajectory. The CORR assessed the similarities in the shapes, RMSE measured the absolute deviation and TD evaluated the time shift between the two trajectories. Importantly, TD corresponds to the time delay between the input and output of the closed-loop control system, and it, therefore, reflects pure delays (perceptual and motor processing) as well as the dynamics (transfer function) of the human controller. Note that TD was computed first by locating the maximum of cross-correlation function, and then CORR and RMSE were calculated using a time-shifted version of the generated trajectory. Therefore, the time shift between the trajectories did not affect the RMSE and CORR. Better quality of tracking is indicated with higher CORR and lower RMSE or TD.

### Data Analysis

The data were not normally distributed (Lilliefors test) and therefore non-parametric tests were used to assess statistically significant differences. To evaluate the effect of training, the Friedman test was applied to compare the outcome measures across trials for each simulation modality. If the test indicated significant differences, *post hoc* pairwise comparison was run using Tukey’s honestly significant difference criterion. To compare the modalities, the average performance for each subject was computed over the last five trials (steady-state) while the first three trials were associated with familiarization and training and the outcome measures were compared between the modalities using the Wilcoxon sign rank test. Additionally, the velocity of joystick movement was computed by low-pass filtering (2nd order Butterworth, 6 Hz cutoff) and then differentiating the generated joystick trajectory. Since the reference trajectory comprised three repeated segments, mean and maximum absolute velocity were computed over these segments of the generated trajectory and averaged across trials for each subject. The obtained results, namely the subject-specific average mean and maximum, were then compared between subdermal and surface conditions using a paired *t*-test, as the Lilliefors test indicated normal distributions. The threshold for the statistical significance was set at *p* < 0.05. The statistical tests have been performed in IBM SPSS version 25 and Matlab, 2020a.

## Results

The stimulation amplitude and pulse width range are listed in Table 1. Note that the pulse amplitude for the subdermal stimulation was from two to three times lower compared to the surface stimulation. The dynamic range of the pulse width was similar between the modalities as well as between the dorsal and volar side of the forearm.

**TABLE 1:** The stimulation amplitude and pulse width range.

**Subjects**	**Surface stimulation**						**Subdermal stimulation**					
	**Dorsal side**			**Volar side**			**Dorsal side**			**Volar side**		
		**Pulse width range (μs)**			**Pulse width range (μs)**			**Pulse width range (μs)**			**Pulse width range (μs)**	
	**Amplitude (mA)**	**Lower**	**Higher**	**Amplitude (mA)**	**Lower**	**Higher**	**Amplitude (mA)**	**Lower**	**Higher**	**Amplitude (mA)**	**Lower**	**Higher**
1	3.5	205	912	3	187	658	1.5	115	443	1	172	612
2	3.5	185	539	2.5	197	511	2	137	697	1	128	622
3	3.5	156	521	3	232	618	1.5	178	909	1	119	665
4	3.5	177	390	3	240	427	1.5	121	399	1	139	371
5	3.5	158	596	3	206	571	1.5	115	635	1	110	398
6	3.5	139	341	3	167	492	1	110	590	0.3	104	353
7	3.5	177	331	2.5	224	440	1	154	802	1	113	620
8	3	220	913	3.5	190	910	0.8	152	624	0.7	110	590
9	3.5	165	552	3	122	354	1	141	682	1	139	741
10	3.5	166	404	3	158	329	1.5	107	486	1	110	560
Mean	3.5	174.80	549.90	2.95	192.30	531.00	1.33	133.00	626.70	0.90	124.40	553.20

The average mean and maximum joystick velocities were similar in the two conditions: 4.6 ± 0.7 au/s (subdermal) versus 4.9 ± 1.2 au/s (surface) for maximum, and 0.46 ± 0.14 au/s (subdermal) versus 0.47 ± 0.13 au/s (surface) for the mean. Hence, the stimulation modality did not affect how fast the subjects manipulated the joystick to respond to the tracking error. Note the large difference between the average mean and maximum values. This reflects the shape of the velocity profile which comprised a sequence of short peaks (fast corrections) separated by periods of slow or no movements. Such profiles were characteristic of both modalities, and therefore imply similar tracking strategies with the two stimulation methods.

**FIGURE 2:**
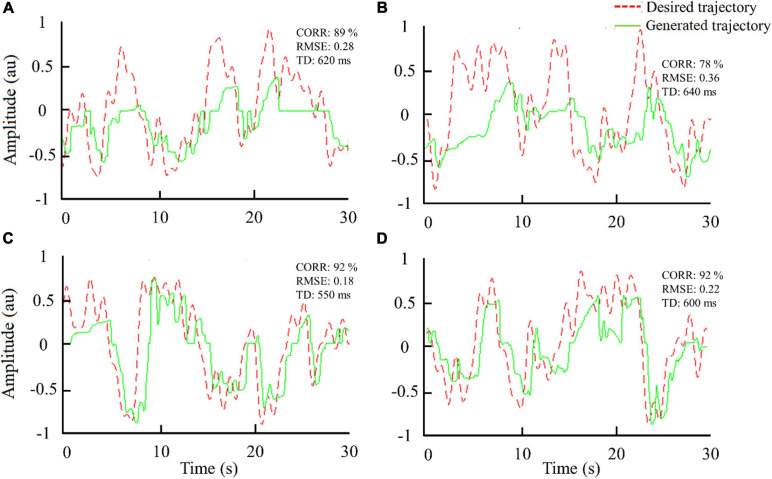
Trajectories generated by one subject during tracking using surface [**(A)** trial 1 and **(C)** trial 8] and subdermal stimulation [**(B)** trial 1 and **(D)** trial 8]. Notation: RMSE, root mean square error; CORR, correlation; TD, time delay.

The representative tracking trajectories generated by one subject with surface and subdermal stimulation in trials 1 and 8 are presented in Figure 2. The CORRs between reference and generated trajectories are 89 and 78% for trials 1 of surface and subdermal stimulation (Figures 2A,B), and 92% for trials 8 of surface and subdermal stimulation (Figures 2C,D), respectively. Note that the subjects improved their performance across trials in both modalities (higher CORR and lower RMSE). Initially, they had trouble tracking the positive segment of the trajectory, and this was especially expressed in subdermal stimulation. Nevertheless, in the final trial, the tracking was good in both directions, and the quality of tracking was better with surface stimulation, where the subject was able to reproduce even small wiggles in the trajectory. In the subdermal condition, on the other side, he/she reproduced only the general trend.

**FIGURE 3:**
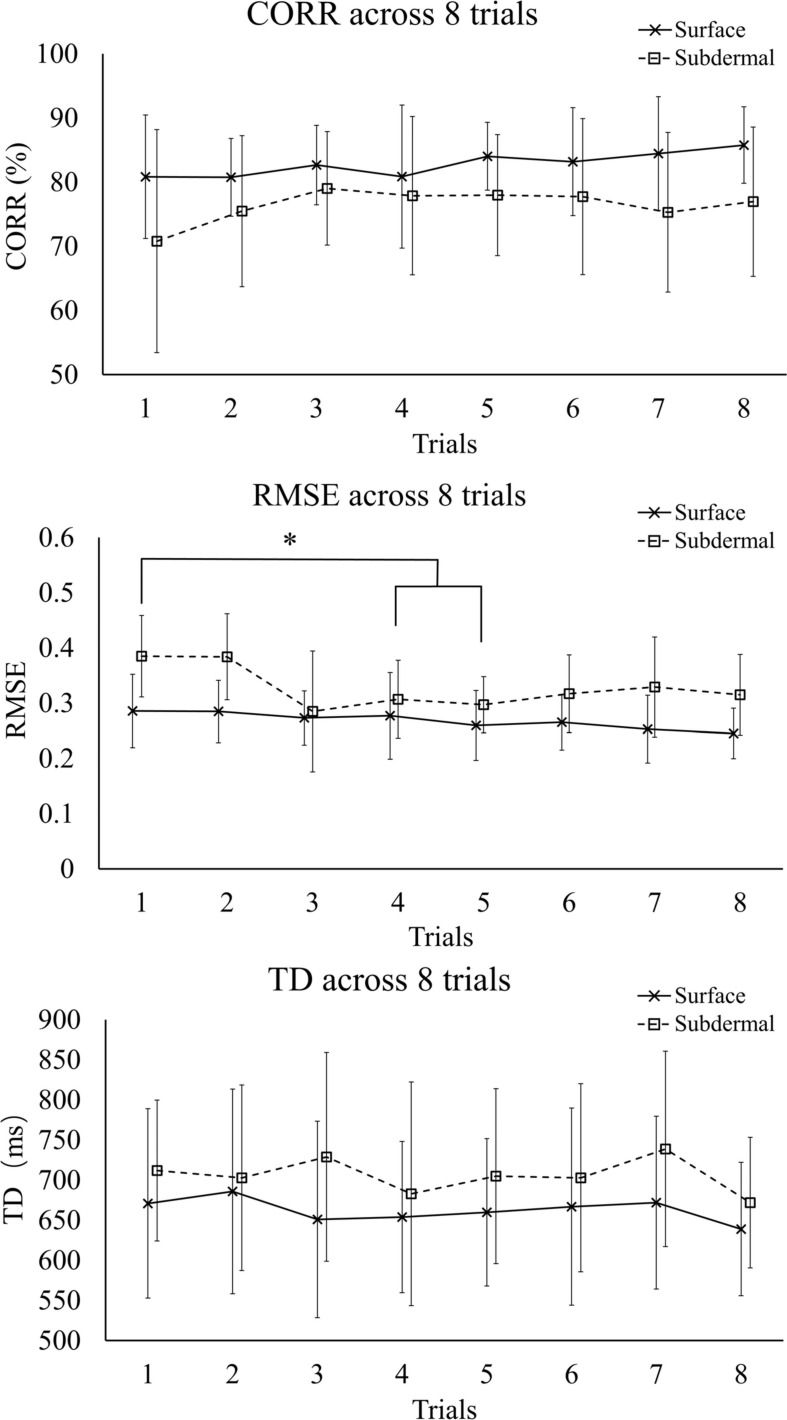
Summary performance (mean ± standard deviation) for CORR, RMSE, and TD across trials. The symbol * indicates a statistically significant difference (*p* < 0.05). RMSE, root mean square error; CORR, correlation; TD, time delay.

The effect of training is summarized in Figure 3, which shows all outcome measures for each of the eight trials averaged across subjects. The trend of increasing performance (decrease in RMSE, increase in CORR) is visible in both modalities. This trend is gradual for the surface stimulation, while in subdermal stimulation, there is a more pronounced increase in the first three trials. And indeed, the linear regression fit indicated statistically significant trend for the decrease in RMSE (*p* < 0.05) with surface stimulation, while the Friedman test revelaed statistically significant (*p* < 0.01) change in performance across trials for the RMSE in subdermal condition. The *post hoc* tests indicated that RMSE decreased significantly from the 1^st^ (0.38 ± 0.07) to 4^th^ (0.3 ± 0.07) and 5^th^ (0.29 ± 0.05) trial.

**FIGURE 4:**
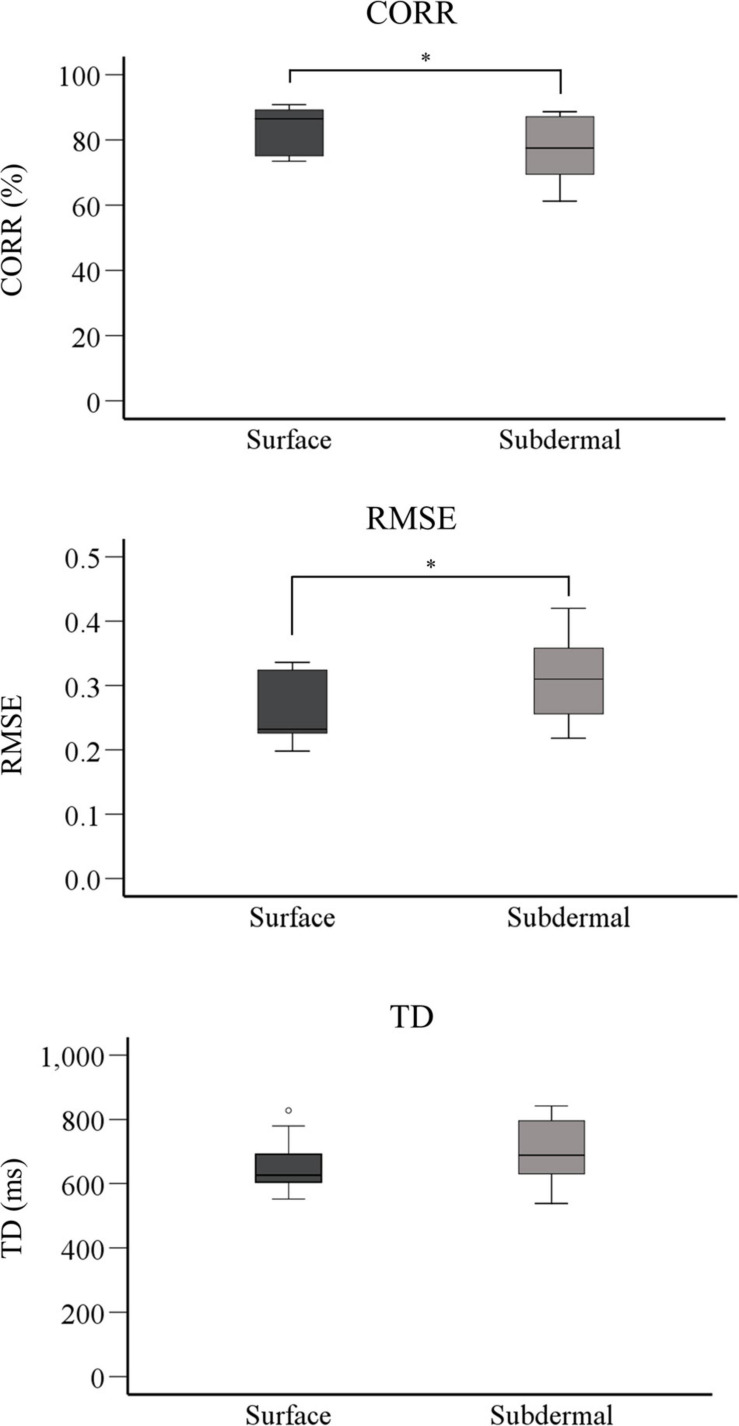
Boxplots showing summary performance for CORR, RMSE, and TD for the last 5 tracking trials (steady-state). The horizontal line is the mean value, the box indicates interquartile range, and whiskers are minimum and maximum values. The symbol * indicates a statistically significant difference (*p* < 0.05). RMSE, root mean square error; CORR, correlation, TD, time delay.

Figure 4 shows a summary performance for the two stimulation modalities averaged across the last 5 trials (steady-state). The surface stimulation significantly outperformed the subdermal tracking in RMSE (*p* < 0.01) and CORR (*p* < 0.05), with median (interquartile range) of 0.23(0.09) versus 0.31(0.10) for RMSE and 86(14%) versus 77(18%) for CORR, respectively. There was no significant difference in TD between the two modalities, although the mean TD of subdermal stimulation was consistently higher across trials (Figure 3). The median (interquartile range) was 626(88) ms for surface and 689(176) ms for subdermal stimulation.

## Discussion

The current study assessed the performance of subdermal and surface stimulation for providing tactile sensory feedback during the online compensatory tracking task. We have selected online compensatory tracking to compare the two interfaces under identical and general conditions, which do not depend on the characteristics of the specific device and/or task. As such, the tracking results cannot be directly related to a prosthesis application. In fact, during the actual prosthesis use, the deviation from the desired goal (the tracking error) will not be available, but instead, the subject will need to estimate this quantity. Nevertheless, online tracking allows an objective comparison of the two interfaces since the results are not affected by the dynamics of the system or task requirements. In the latter case, the results can be specific to the context in which the test has been performed. For instance, the feedback modalities can perform similarly with one prosthesis or for a particular number of force levels, whereas the difference can be significant if the number of force levels is increased or if the prosthesis is changed (from slower to faster). Finally, compensatory tracking was used routinely in the past for the evaluation of human manual control (of which prosthesis use is one example) to investigate the characteristics of both visual (McRuer and Krendel, 1959; Mcruer and Weir, 1969) and electrotactile feedback (Schori, 1970; Schmid and Bekey, 1978; Paredes et al., 2015).

The results demonstrated that surface stimulation resulted in a better quality of tracking as demonstrated by a significantly higher CORR and lower RMSE compared to subdermal stimulation. The surface stimulation decreased RMSE and increased CORR for approximately 33% and 10%, respectively. In our recent study (Dong et al., 2020b), we have demonstrated that the surface stimulation leads to a higher number of discriminable levels of tactile sensations that can be elicited between the detection and pain thresholds. The present study suggests that higher psychometric resolution leads to better closed-loop control. Nevertheless, both stimulation modalities led to a good quality of closed-loop control (see Figures 2, 3, CORR > 75%) and the subdermal stimulation has some important advantages for practical application, as discussed below. Also, there was no significant difference in TD between the two stimulation modalities, which indicated that the reaction time after perceiving surface and subdermal stimulation was similar. Finally, there is a trend for the effects of training in both modalities (see Figure 3, RMSE panel).

Interestingly, the improvement in performance for subdermal stimulation is especially expressed in the first three trials. We hypothesize that the training is more dominant in the subdermal stimulation due to the more invasive nature of the interface. The stimulation is delivered below the skin and the subjects used initial trials to adapt to the new sensation. This might have been due to physiological factors (e.g., different recruitment of mechanoreceptors) and/or purely psychological effects (e.g., anxiety due to invasiveness). The same factors could have contributed to the general difference in performance between the two modalities. An advantage of subdermal stimulation is that it could bypass the scar tissue at the residual limb. This depends on the condition of the skin (scar tissue depth). The subdermal electrode can be inserted into the intact tissue beneath the scar, thereby potentially improving the sensitivity. We expect that similar conclusions, as in the present study, would be obtained if the electrodes would be applied to a different location or even a different segment, as long as both electrode types would be applied to the same skin area.

Practically, the long-term use of the stimulation modalities for providing sensory feedback should be considered. The advantage of subdermal stimulation is that it leads to significantly lower power consumption (see Table 1). In addition, the subdermal interface can be designed and manufactured as an implantable electrode with stretchable materials (Musick et al., 2015; Thompson et al., 2016), which could endure the local tissue stretching during body movements and long-term implantation. However, the subdermal interface in any form is still a minimally invasive solution with the risk of infections and some pain during insertion and removal, especially in case of percutaneous approach, as well as unclear long-term stability (months to years), and possible complications after long-term implantation. Nevertheless, a recent study demonstrated a successful chronic application of a novel button-type percutaneous electrode for EMG recording (Hahne et al., 2016). Finally, the subdermal electrodes could be used as a fully implanted solution, where both the stimulation electronics and electrode pads are placed just below the skin.

A simple linear encoding has been used in the present study to map the feedback variable into stimulation intensity. Another encoding could affect the quality of tracking (Szeto, 1977), but as long as the same encoding is used for both stimulation modalities, we assume that this would not affect the relative performance (comparison). Furthermore, a unique advantage of electrotactile interface is that the stimulation parameters (intensity and frequency) can be changed independently. Therefore, it would be interesting to test the quality of online control when using frequency modulation and compare it to that achieved with pulse width modulation (present experiment), particularly since recent studies have reported conflicting results (Valle et al., 2018b; Dideriksen et al., 2020). The two schemes can be even combined [i.e., simultaneous change in amplitude and frequency (George et al., 2019)] to exploit the full range of sensations. And indeed, different coding schemes might change the relative performance of the two stimulation modalities, and this needs to be investigated in future studies.

Overall, both modalities result in good closed-loop control, and the subdermal stimulation has potential advantages concerning applicability. Therefore, the latter method can be a promising technique to provide sensory feedback in a prosthesis. In our previous work, we have investigated “static” psychometric properties of subdermal stimulation (Geng et al., 2018), assessed their stability within hours (Dong et al., 2020b) and days (Dong et al., 2020a), and in the present study, we have evaluated this method within an online control loop. These results provide relevant knowledge for the application of subdermal stimulation in amputee subjects using a sensate prosthesis, which is indeed the next step in our research. In this case, it would be relevant to compare the performance with and without tactile feedback to assess the added value of the subdermal interface. As reported in a recent review (Sensinger and Dosen, 2020), both non-invasive and invasive approaches to restoring sensory feedback can improve performance and user experience, yet this is not a simple relation but depends on many factors. The functional test will allow comparing potential benefits to the challenges related to the application of the subdermal interface (e.g., minimal invasiveness). Also, a multichannel subdermal interface should be designed and tested to truly exploit the benefits of this technique (e.g., compactness). Since the electrode is small (a tip of a wire), many channels could be easily positioned closed to each other in an array or a matrix configuration.

## Data Availability Statement

The original contributions presented in the study are included in the article, further inquiries can be directed to the corresponding author.

## Ethics Statement

The studies involving human participants were reviewed and approved by North Denmark Region Committee on Health Research Ethics (N-20160021). The patients/participants provided their written informed consent to participate in this study.

## Author Contributions

JD, BG, EK, and SD contributed to experimental design and development. JD, WJ, EK, and SD performed the experimental data collection and data analysis. JD, BG, and SD coordinated with participants and were the principal investigators. All authors contributed to the manuscript preparation and writing.

## Conflict of Interest

The authors declare that the research was conducted in the absence of any commercial or financial relationships that could be construed as a potential conflict of interest.

## References

[B1] Biddiss E. Beaton D. Chau T. (2007). Consumer design priorities for upper limb prosthetics. *Disabil. Rehabil. Assist. Technol.* 2 346–357. 10.1080/17483100701714733 19263565

[B2] Chadwell A. Kenney L. Thies S. Galpin A. Head J. (2016). The reality of myoelectric prostheses: Understanding what makes these devices difficult for some users to control. *Front. Neurorobot.* 10:7. 10.3389/fnbot.2016.00007 27597823 PMC4992705

[B3] Clemente F. D’Alonzo M. Controzzi M. Edin B. Cipriani C. (2016). Non-invasive, temporally discrete feedback of object contact and release improves grasp control of closed-loop myoelectric transradial prostheses. *IEEE Trans. Neural Syst. Rehabil. Eng.* 24 1314–1322. 10.1109/tnsre.2015.2500586 26584497

[B4] Clemente F. Valle G. Controzzi M. Strauss I. Iberite F. Stieglitz T. (2019). Intraneural sensory feedback restores grip force control and motor coordination while using a prosthetic hand. *J Neural Eng.* 16 026034. 10.1088/1741-2552/ab059b 30736030

[B5] D’Anna E. Petrini F. M. Artoni F. Popovic I. Simaniæ I. Raspopovic R. (2017). A somatotopic bidirectional hand prosthesis with transcutaneous electrical nerve stimulation based sensory feedback. *Sci Rep* 7 10930. 10.1038/s41598-017-11306-w 28883640 PMC5589952

[B6] De Nunzio A. M. Dosen S. Lemling S. Markovic M. Schweisfurth M. A. Ge N. (2017). Tactile feedback is an effective instrument for the training of grasping with a prosthesis at low- and medium-force levels. *Exp. Brain Res.* 235 2547–2559. 10.1007/s00221-017-4991-7 28550423 PMC5502062

[B7] Dideriksen J. L. Mercader I. Dosen S. (2020). Closed-loop Control using Electrotactile Feedback Encoded in Frequency and Pulse Width. *IEEE Trans. Haptics* 13 818–824. 10.1109/TOH.2020.2985962 32287006

[B8] Dong J. Geng B. Niazi I. K. Amjad I. Dosen S. Jensen W. (2020a). The Variability of Psychophysical Parameters following Surface and Subdermal Stimulation: A Multiday Study in Amputees. *IEEE Trans. Neural Syst. Rehabil. Eng.* 28 174–180. 10.1109/TNSRE.2019.2956836 31796411

[B9] Dong J. Kamavuako E. N. Dosen S. Jensen W. Geng B. (2020b). The Short-Term Repeatability of Subdermal Electrical Stimulation for Sensory Feedback. *IEEE Access* 8 63983–63992. 10.1109/ACCESS.2020.2984534

[B10] Dosen S. Markovic M. Hartmann C. Farina D. (2015). Sensory feedback in prosthetics: a standardized test bench for closed-loop control. *IEEE Trans. Neural Syst. Rehabil. Eng.* 23 267–276. 10.1109/TNSRE.2014.2371238 25420268

[B11] Dosen S. Schaeffer M.-C. Farina D. (2014). Time-division multiplexing for myoelectric closed-loop control using electrotactile feedback. *J. Neuroeng. Rehabil.* 11 1–10. 10.1186/1743-0003-11-138 25224266 PMC4182789

[B12] Engels L. F. Shehata A. W. Scheme E. J. Sensinger J. W. Cipriani C. (2019). When Less Is More – Discrete Tactile Feedback Dominates Continuous Audio Biofeedback in the Integrated Percept While Controlling a Myoelectric Prosthetic Hand. *Front. Neurosci.* 13:578. 10.3389/fnins.2019.00578 31244596 PMC6563774

[B13] Fougner A. Stavdahl O. Kyberd P. J. Losier Y. G. Parker P. A. (2012). Control of upper limb prostheses: Terminology and proportional myoelectric controla review. *IEEE Trans. Neural Syst. Rehabil. Eng.* 20 663–677. 10.1109/TNSRE.2012.2196711 22665514

[B14] Geng B. Dong J. Jensen W. Dosen S. Farina D. Kamavuako E. N. (2018). Psychophysical evaluation of subdermal electrical stimulation in relation to prosthesis sensory feedback. *IEEE Trans. Neural Syst. Rehabil. Eng.* 26 709–715. 10.1109/TNSRE.2018.2803844 29522414

[B15] George J. A. Kluger D. T. Davis T. S. Wendelken S. M. Okorokova E. V. He Q. (2019). Biomimetic sensory feedback through peripheral nerve stimulation improves dexterous use of a bionic hand. *Sci. Robot.* 4 1–11. 10.1126/scirobotics.aax2352 33137773

[B16] Graczyk E. L. Resnik L. Schiefer M. A. Schmitt M. S. Tyler D. J. (2018). Home use of a neural-connected sensory prosthesis provides the functional and psychosocial experience of having a hand again. *Sci. Rep.* 8 1–17. 10.1038/s41598-018-26952-x 29959334 PMC6026118

[B17] Hahne J. M. Farina D. Jiang N. Liebetanz D. (2016). A Novel Percutaneous Electrode Implant for Improving Robustness in Advanced Myoelectric Control. *Front. Neurosci.* 10:114. 10.3389/fnins.2016.00114 27065783 PMC4814550

[B20] Kingdom F. A. A. Prins N. (2009). *Psychophysics: A Practical Introduction*, 1st Edn. Cambridge, MA: Academic Press.

[B21] Kyberd P. J. Wartenberg C. Sandsjö L. Jönsson S. Gow D. Frid J. (2007). Survey of upper-extremity prosthesis users in Sweden and the United Kingdom. *J. Prosthetics Orthot.* 19 55–62. 10.1097/JPO.0b013e3180459df6

[B23] LUKE arm. (2020). *Clinical relevance.* Available online at: https://www.mobiusbionics.com/luke-arm/#section-four (Accessed Oct. 29, 2020)

[B24] Markovic M. Schweisfurth M. A. Engels L. F. Bentz T. Wüstefeld D. Farina D. (2018). The clinical relevance of advanced artificial feedback in the control of a multi-functional myoelectric prosthesis. *J. Neuroeng. Rehabil.* 15 1–15. 10.1186/s12984-018-0371-1 29580245 PMC5870217

[B25] Mastinu E. Engels L. F. Clemente F. Dione M. Sassu P. Aszmann O. (2020). Neural feedback strategies to improve grasping coordination in neuromusculoskeletal prostheses. *Sci. Rep.* 10 11793. 10.1038/s41598-020-67985-5 32678121 PMC7367346

[B26] Mcruer D. Weir D. H. (1969). Theory of Manual Vehicular Control. *Ergonomics* 12 599–633. 10.1080/00140136908931082 5823971

[B27] McRuer D. T. Krendel E. S. (1959). The human operator as a servo system element. *J. Franklin Inst.* 267 381–403. 10.1016/0016-0032(59)90091-2

[B28] Musick K. M. Rigosa J. Narasimhan S. Wurth S. Capogrosso M. Chew D. J. (2015). Chronic multichannel neural recordings from soft regenerative microchannel electrodes during gait. *Nat. Publ. Gr.* 5 1–9. 10.1038/srep14363 26400791 PMC4585830

[B29] Oddo C. M. Raspopovic S. Artoni F. Mazzoni A. Spigler G. Petrini F. (2016). Intraneural stimulation elicits discrimination of textural features by artificial fingertip in intact and amputee humans. *Elife* 5 1–27. 10.7554/eLife.09148 26952132 PMC4798967

[B30] Osborn L. E. Dragomir A. Betthauser J. L. Hunt C. T. Nguyen H. H. Kaliki R. R. (2018). Prosthesis with neuromorphic multilayered e-dermis perceives touch and pain. *Sci Robot* 3 eaat3818. 10.1126/scirobotics.aat3818 32123782 PMC7051004

[B31] Ossur. (2020). *Ossur i-Limb§*. Available online at: https://www.ossur.com/en-us/prosthetics/arms/i-limb-ultra-titanium (Accessed Oct. 29, 2020)

[B32] Ottobock. (2020a). *Ottobock bebionic hand.* Available online at: https://www.ottobockus.com/prosthetics/upper-limb-prosthetics/solution-overview/bebionic-hand/ (Accessed Oct. 29, 2020a)

[B33] Ottobock. (2020b). *Ottobock SensorHand Speed.* Available online at: https://www.ottobockus.com/prosthetics/upper-limb-prosthetics/solution-overview/myoelectric-devices-speedhands/index.html (Accessed Oct. 29, 2020b)

[B34] Page D. M. George J. A. Kluger D. T. Duncan C. Wendelken S. Davis T. (2018). Motor Control and Sensory Feedback Enhance Prosthesis Embodiment and Reduce Phantom Pain After Long-Term Hand Amputation. *Frontiers in human neuroscience* 12:352. 10.3389/fnhum.2018.00352 30319374 PMC6166773

[B35] Paredes L. P. Dosen S. Rattay F. Graimann B. Farina D. (2015). The impact of the stimulation frequency on closed-loop control with electrotactile feedback. *J. Neuroeng. Rehabil.* 12 1–16. 10.1186/s12984-015-0022-8 25889752 PMC4403675

[B36] Parker P. Englehart K. Hudgins B. (2006). Myoelectric signal processing for control of powered limb prostheses. *J. Electromyogr. Kinesiol.* 16 541–548. 10.1016/j.jelekin.2006.08.006 17045489

[B37] Pena A. E. Rincon-Gonzalez L. Abbas J. J. Jung R. (2019). Effects of vibrotactile feedback and grasp interface compliance on perception and control of a sensorized myoelectric hand. *PLoS One* 14:e0210956. 10.1371/journal.pone.0210956 30650161 PMC6334959

[B38] Petrini F. M. Valle G. Strauss I. Granata G. Di Iorio R. D’Anna E. (2019). Six-Month Assessment of a Hand Prosthesis with Intraneural Tactile Feedback. *Annals of neurology* 85 137–154. 10.1002/ana.25384 30474259

[B39] Psyonic Ability Hand. (2020). *Psyonic The Ability Hand^TM^*. Available online at: https://www.psyonic.co/abilityhand (Accessed Oct. 29, 2020)

[B40] Raspopovic S. Capogrosso M. Petrini F. M. Bonizzato M. Rigosa J. Di Pino G. (2014). Restoring natural sensory feedback in real-time bidirectional hand prostheses. *Sci. Transl. Med.* 6 222ra19. 10.1126/scitranslmed.3006820 24500407

[B41] Riso R. Ignagni A. R. Keith M. W. (1989). Electrocutaneous Sensations Elicited using Subdermally Located Electrodes. *Automedica* 11 25–42.

[B42] Risso G. Valle G. Iberite F. Strauss I. Stieglitz T. Controzzi M. (2019). Optimal integration of intraneural somatosensory feedback with visual information: a single-case study. *Scientific reports* 9 7916. 10.1038/s41598-019-43815-1 31133637 PMC6536542

[B43] Rognini G. Petrini F. M. Raspopovic S. Valle G. Granata G. Strauss I. (2019). Multisensory bionic limb to achieve prosthesis embodiment and reduce distorted phantom limb perceptions. *Journal of neurology, neurosurgery, and psychiatry* 90 833–836. 10.1136/jnnp-2018-318570 30100550 PMC6791810

[B45] Schiefer M. Tan D. Sidek S. M. Tyler D. J. (2016). Sensory feedback by peripheral nerve stimulation improves task performance in individuals with upper limb loss using a myoelectric prosthesis. *Journal of neural engineering* 13 016001. 10.1088/1741-2560/13/1/016001 PMC5517302 26643802

[B46] Schmid H. P. Bekey G. A. (1978). Tactile Information Processing by Human Operators in Control Systems. *IEEE Trans. Syst. Man Cybern.* 8 860–866. 10.1109/TSMC.1978.4309886

[B48] Schoepp K. R. Dawson M. R. Schofield J. S. Carey J. P. Hebert J. S. (2018). Design and integration of an inexpensive wearable mechanotactile feedback system for myoelectric prostheses. *IEEE J. Transl. Eng. Heal. Med.* 6 1–11. 10.1109/JTEHM.2018.2866105 30197843 PMC6126793

[B49] Schori T. R. (1970). Tracking Performance as a Function of Precision of Electrocutaneous Feedback Information. *Hum. Factors J. Hum. Factors Ergon. Soc.* 12 447–452. 10.1177/001872087001200503

[B50] Sensinger J. W. Dosen S. (2020). A Review of Sensory Feedback in Upper-Limb Prostheses From the Perspective of Human Motor Control. *Front. Neurosci.* 14:345. 10.3389/fnins.2020.00345 32655344 PMC7324654

[B51] Shehata A. W. Engels L. F. Controzzi M. Cipriani C. Scheme E. J. Sensinger J. W. (2018). Improving internal model strength and performance of prosthetic hands using augmented feedback. *J. Neuroeng. Rehabil.* 15 1–12. 10.1186/s12984-018-0417-4 30064477 PMC6069837

[B52] Shin H. Watkins Z. Huang H. H. Zhu Y. Hu X. (2018). Evoked haptic sensations in the hand via non-invasive proximal nerve stimulation. *Journal of neural engineering* 15 046005. 10.1088/1741-2552/aabd5d 29638220

[B53] Szeto A. Y. (1977). Comparison of codes for sensory feedback using electrocutaneous tracking. *Annals of biomedical engineering* 5 367–383. 10.1007/BF02367316 607824

[B54] Szeto A. Y. J. Saunders F. A. (1982). Electrocutaneous Stimulation for Sensory Communication in Rehabilitation Engineering. *IEEE Trans. Biomed. Eng. BME-* 29 300–308. 10.1109/TBME.1982.324948 7068167

[B55] Tabot G. A. Dammann J. F. Berg J. A. Tenore F. V. Boback J. L. Vogelstein R. J. (2013). Restoring the sense of touch with a prosthetic hand through a brain interface. *Proc. Natl. Acad. Sci. U. S. A.* 110 18279–18284. 10.1073/pnas.1221113110 24127595 PMC3831459

[B56] Tan D. W. Schiefer M. A. Keith M. W. Anderson J. R. Tyler D. J. (2015). Stability and selectivity of a chronic, multi-contact cuff electrode for sensory stimulation in human amputees. *J. Neural Eng.* 12 1–10. 10.1088/1741-2560/12/2/026002 PMC5517311 25627310

[B57] Thompson C. H. Zoratti M. J. Langhals N. B. Purcell E. K. (2016). Regenerative Electrode Interfaces for Neural Prostheses. *Tissue Eng. - Part B Rev.* 22 125–135. 10.1089/ten.teb.2015.0279 26421660

[B58] Valle G. D’Anna E. Strauss I. Clemente F. Granata G. Di Iorio R. (2020). Hand Control With Invasive Feedback Is Not Impaired by Increased Cognitive Load. *Front. Bioeng. Biotechnol.* 8:287. 10.3389/fbioe.2020.00287 32318562 PMC7147827

[B59] Valle G. Mazzoni A. Iberite F. D’Anna E. Strauss I. Granata G. (2018a). Biomimetic Intraneural Sensory Feedback Enhances Sensation Naturalness, Tactile Sensitivity, and Manual Dexterity in a Bidirectional Prosthesis. *Neuron* 100 37.e–45.e. 10.1016/j.neuron.2018.08.033 ^∗∗^37-45.e7 30244887

[B60] Valle G. Petrini F. M. Strauss I. Iberite F. D’Anna E. Granata G. (2018b). Comparison of linear frequency and amplitude modulation for intraneural sensory feedback in bidirectional hand prostheses. *Scientific reports* 8 16666. 10.1038/s41598-018-34910-w 30420739 PMC6232130

[B62] VINCENTevolution 2 (2020). *Vincent Systems VINCENT EVOLUTION 2.* Available online at: https://vincentsystems.de/en/prosthetics/vincent-evolution-2/ (Accessed Oct. 29, 2020).

[B63] Vujaklija I. Farina D. Aszmann O. (2016). New developments in prosthetic arm systems. *Orthop. Res. Rev.* 8 31–39. 10.2147/ORR.S71468 30774468 PMC6209370

[B64] Wheeler J. Bark K. Savall J. Cutkosky M. (2010). Investigation of rotational skin stretch for proprioceptive feedback with application to myoelectric systems. *IEEE Trans. Neural Syst. Rehabil. Eng.* 18 58–66. 10.1109/TNSRE.2009.2039602 20071271

[B65] Witteveen H. J. B. Rietman H. S. Veltink P. H. (2015). Vibrotactile grasping force and hand aperture feedback for myoelectric forearm prosthesis users. *Prosthet. Orthot. Int.* 39 204–212. 10.1177/0309364614522260 24567348

[B66] Zollo L. Di Pino G. Ciancio A. L. Ranieri F. Cordella F. Gentile C. (2019). Restoring tactile sensations via neural interfaces for real-time force-and-slippage closed-loop control of bionic hands. *Sci. Robot.* 4 1–28. 10.1126/scirobotics.aau9924 31620665 PMC6795534

